# To Procrastinate or Not to Procrastinate: A Retrospective Study of the Optimal Timing of Containing the Global Spread of the COVID-19 Pandemic

**DOI:** 10.3389/fpubh.2021.613980

**Published:** 2021-08-03

**Authors:** Jun Li, Lingjian Ye, Yimin Zhou, Joy Y. Zhang, Zhuo Chen

**Affiliations:** ^1^School of International Relations, Sun Yat-sen University, Guangzhou, China; ^2^School of Management, Curtin University of Technology, Perth, WA, Australia; ^3^Shenzhen Institute of Advanced Technology, Chinese Academy of Sciences, Shenzhen, China; ^4^University of Chinese Academy of Sciences, Beijing, China; ^5^School of Social Policy, Sociology and Social Research, University of Kent, Canterbury, United Kingdom; ^6^Institute of Communication and Global Public Opinion, Xi'an International Studies University, Xi'an, China

**Keywords:** COVID-19 pandemic, prevention and control, second wave outbreak, timing, optimal control, irreversible damage, retrospective study

## Abstract

As global public health is under threat by the 2019-nCoV and a potential new wave of large-scale epidemic outbreak and spread is looming, an imminent question to ask is what the optimal strategy of epidemic prevention and control (P&C) measures would be, especially in terms of the timing of enforcing aggressive policy response so as to maximize health efficacy and to contain pandemic spread. Based on the current global pandemic statistic data, here we developed a logistic probability function configured SEIR model to analyse the COVID-19 outbreak and estimate its transmission pattern under different “anticipate- or delay-to-activate” policy response scenarios in containing the pandemic. We found that the potential positive effects of stringent pandemic P&C measures would be almost canceled out in case of significantly delayed action, whereas a partially procrastinatory wait-and-see control policy may still be able to contribute to containing the degree of epidemic spread although its effectiveness may be significantly compromised compared to a scenario of early intervention coupled with stringent P&C measures. A *laissez-faire* policy adopted by the government and health authority to tackling the uncertainly of COVID19-type pandemic development during the early stage of the outbreak turns out to be a high risk strategy from optimal control perspective, as significant damages would be produced as a consequence.

## Introduction

The COVID-19 pandemic has led to serious socioeconomic consequences and could generate far-reaching impacts on global public health governance^1^. Mitigating and containing the further spread of COVID-19 has become a top priority in the international community. It is widely accepted in the epidemic research scholarship that the early stage of epidemic development is a crucial period for effectively containing virus spread over the global community, thus a good understanding of how the pandemic has been developed into a world concern as well as its progression course allows both health researchers and policy makers to review and re-assess epidemic control strategy in a timely manner in order to improve the local and global responsiveness facing the potential threats. However, a majority of the existing quantitative epidemiological studies have primarily focused on forecasting the coronavirus's future evolution scenarios, while few of them has attempted to explain the mechanisms underlying the course along which the COVID-19 crisis has unfolded and developed into such an overwhelming scale that global health institutions still struggle to tackle it so far. To address this research gap, there is an urgent need to go backward to the early stage of the coronavirus outbreak. It is of particular importance in understanding how the initial sparsely distributed local epidemic events have eventually developed into a global pandemic. Thus it is necessary to explore different courses of potential spread of country-wise COVID-19 epidemic dynamics from a retrospective standpoint.

Based on a retrospective investigation of epidemic growth, this study aims to build a new hybrid epidemic modeling framework to facilitate our understanding of which key variables may exercise significant influences in simulating and forecasting different scenarios of COVID-19 infections across a number of representative countries. Our study time span covers the first phase of the epidemic development, i.e., from the initial novel coronavirus outbreak in late January through to the end of March 2020 when the pandemic had rampaged through the majority of the world's populous nations.

According to WHO (2020-3-25), 136 countries have implemented measures that significantly intervened international traffic as defined under Article 43 of the International Health Regulations (2005) as of March 25. This pandemic has posed a daunting challenge to global health governance (1, 2), especially for the lower income countries (3). The uncontrolled development during the early phase (until the middle of April 2020) of the epidemic has made it clear that the novel coronavirus COVID-19, which first broke out in China, and its global spread is a rapidly evolving situation (4–7). Over 162 million cases were confirmed in the world as of May 15, 2021, affecting 219 countries and regions as well as 2 international conveyances, causing over 3.3 million deaths (8). The pandemic has been sweeping all continents. For the time being, Europe and the US have been particularly struck by the pandemic and have the largest number of infected (9). Across the Atlantic, the US has now the largest number of confirmed cases in the world and the situation has sharply deteriorated over the last 2 weeks. On the other hand, many developing countries and lower income nations are particularly vulnerable and may face serious humanitarian crises in the case of unconstrained cross-globe spread, especially in Africa, due to underdeveloped local public health infrastructure (10).

**Figure 1:**
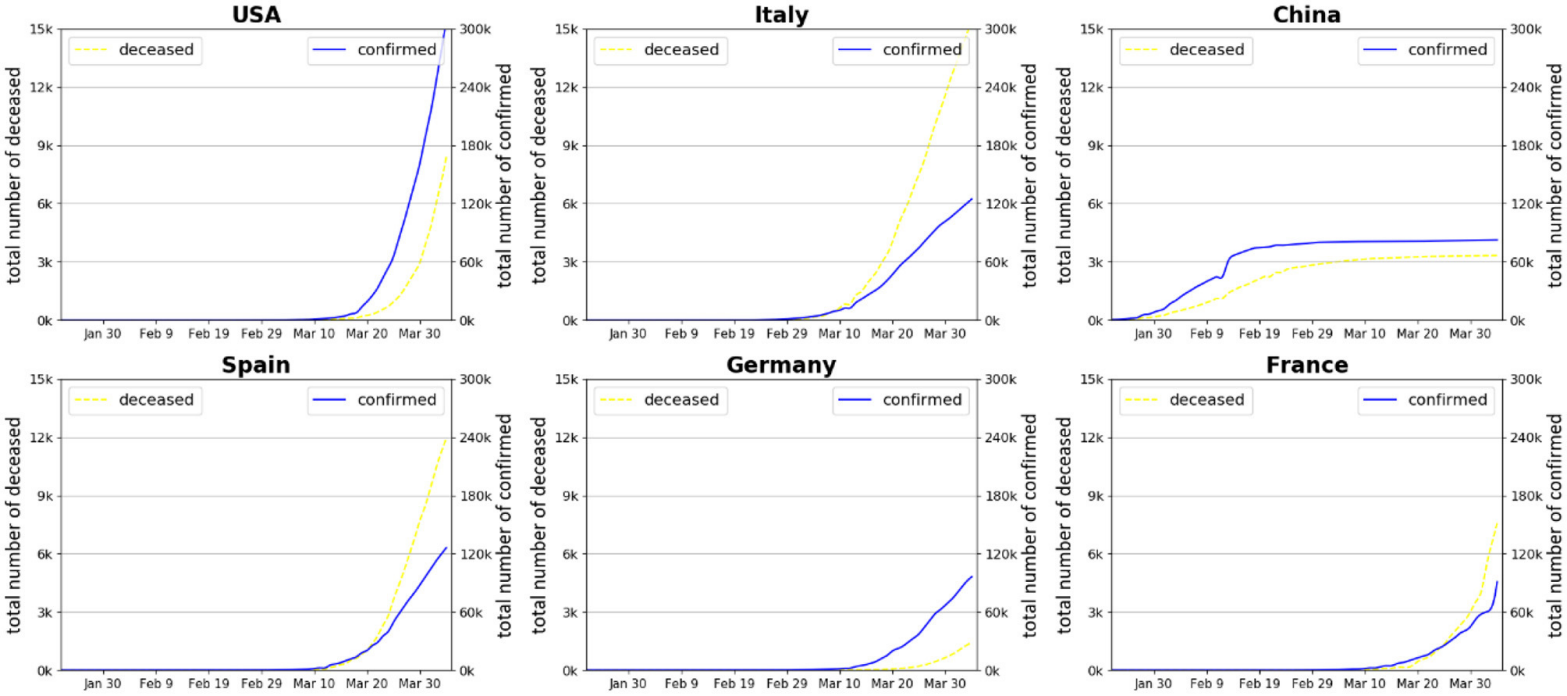
Number of confirmed cases and deceased patients in six countries during the early stage of COVID-19 pandemic development. Source: World Health Organization (14).

In several recent studies, scholars have found solid evidence that people can be reinfected with the virus that causes COVID-19 (Kupferschmidt, *Science*, 2020; Rasmussen, *Clinical Infectious Diseases* 2020^2^), global health may face the new risk of a large-scale second wave of virus infection while the pandemic has entered a new development phase. In order to attain the target of efficient containment of the epidemic and to prevent a likely second-wave global outbreak, especially in countries and regions where the initial infections have been brought under control to more or less stabilization, a central question that remains to be addressed by national and transnational governments is how to take the *time* effect into pandemic control when introducing and enforcing national responses based on previous experiences learned through the global collaboration in combatting COVID-19 (11–13). Thus from a global health governance perspective, it is necessary to conduct a retrospective investigation to examine how different control and prevention measures in the early stage of the pandemic's outbreak may lead to markedly different later contamination pathways. Figure 1 delineates the dynamics of total confirmed cases in six main countries (US, China, Italy, Spain, Germany, and France) that have been seriously affected by COVID-19 since its outbreak up to date. It shows that the situation started to deteriorate rapidly with the number of confirmed cases rising exponentially in European countries and in the US since the middle of March, as a direct result of a lack of preparedness (NYT, 2020^3^). Afterwards, most new cases have been confirmed in Europe and the US. By contrast, the situation has been under control with only a small number of new confirmed cases in China since early March due to the Chinese government's draconian quarantine policy and transmission control measures implemented throughout the entire country^4^. The curve of Chinese confirmed cases has remained flat after reaching a plateau around early March.

The question is as to when to activate aggressive policy responses such as closing of public transport, mandatory self-quarantine, social distancing, and diversion of production to maximize the national health system's efficacy to help contain pandemic spread. Figure 1 implies that the timing of adopting strict prevention and control (P&C) measures is crucial in shaping the national curves of spread and affection of COVID-19. Delaying the stringent P&C measures would risk exacerbating the peak of mass infections which would exhaust the national health system's maximum capacity, putting the medical system on the verge of collapse. However, a review of previous literature suggests that the effectiveness and optimality of timing of COVID-19 pandemic P&C measures remain unknown (8, 15, 16).

A common feature of severely affected countries across Europe and the US in dealing with the COVID-19 pandemic is that a “wait and see” -style containment strategy was adopted by their national or federal governments at the initial stage of the epidemic outbreak, and the public was then not sufficiently advised of taking immediately necessary prevention measures such as self-protection (e.g., wearing face masks when going out), home-isolation, and social distancing by national health authorities, whereas more stringent and radical policies such as city and nationwide lockdown, internal traffic restriction, and outbound/inbound travel ban, were only launched to contain the spread when the pandemic situation had already passed a critical point which accelerated interpersonal transmission and spread of the epidemic with national health system's capacity facing serious saturation challenges and domestic medical resources being rapidly exhausted. Unconventional control and prevention policies such as lockdown and strengthened confinement policies has been continued to be generalized through all European countries since the serious outbreak in Italy around mid-February. Table 1 summarizes major countermeasure milestones of national government responses to containing COVID-19 in the most severely impacted countries together with the latest statistics of infection and death since the epidemic outbreak.

**Table 1:** Milestones of COVID-19 containment policy and virus spread situation in major affected countries (as of April 8, 2020).

**Country**	**Control and prevention measures**	**Total confirmed cases**	**Total deaths**
USA	On March 1, the “right of diagnosis” was opened to improve the timeliness of diagnosis; On March 13, the President declared a state of emergency; On March 14, the U.S. House of Representatives passed a bill to start testing for the novel coronavirus free of charge.	404,242	12,993
Spain	On March 14, Spain closed the country to all citizens except those who had to buy necessities and travel due to uncontrollable factors; On March 16, the government requisitioned protective equipment and equipment from medical institutions across the country; From March 29, all workers in non-core industries were told to stay at home and not go out to work.	146,690	14,555
Italy	On February 24, a number of cities in the north of the country closed, schools closed, carnivals and other activities were canceled; On March 10, a nationwide lockdown was imposed on the closure of cities; On March 20, the first makeshift hospital started operation.	135,586	17,127
Germany	On March 14, 16 federal states closed schools; On March 22, Germany closed its borders, banned gatherings, and put an end to unproductive recreation.	109,329	2,096
France	On March 13, the government banned gatherings of more than 100 people; On March 15, closure of non-essential public places; On March 16, all schools in France were closed; On March 25, a stricter confinement rule was announced, time of outings was restricted to a maximum of 1 h, alone, and once a day.	109,069	10,328
China	On January 23, Wuhan city was locked down; On January 25, 30 provinces launched a public health emergency level I, traffic control was implemented across the whole country; On February 2, “Vulcan mountain” hospital, which is dedicated to treating COVID-19 patients, was put into use; On February 5, Wuhan classified and centralized treatment and isolation of COVID-19 patients; On March 4, a number of provinces and cities introduced measures to strictly prevent overseas imports.	81,802	3,333
Iran	From March 23, schools and other public places were closed, cultural events were canceled, and public transport began to be disinfected daily; On March 25, rules were introduced to restrict travel.	64,586	3,993
UK	On March 3, suspending school classes was suggested by PM; On March 23, the government recommended home quarantine.	60,733	7,097

*Source: WHO Novel Coronavirus disease (COVID-19) Situation report−78*.

*Sciences Po COVID-19 database, https://boogheta.github.io/coronavirus-countries/*

*Worldometer, https://www.worldometers.info/coronavirus/ (accessed April 8, 2020)*.

*Country's P&C measures are referenced in national government's epidemic control policies as detailed at the end of the manuscript*.

The effectiveness of stringent P&C measures is highly influenced by the public authority's degree of epidemic monitoring and enhanced management of the health system. Another critical factor is related to the degree of public compliance (17), which largely relies on how the quarantine measures are enforced by the government. This was particularly relevant during the early stage of the epidemic outbreak in many European countries. Even after strict national lockdown and confinement policies had been announced by national government, there lacked general strict implementation of social distancing in countries such as Italy and France, as people subjected to confinement zones were still allowed outdoors by possession of a travel permit or justification such as going to work or daily shopping needs. In our previous paper, it was shown clearly that the likelihood of success of containing COVID-19 is highly dependent upon whether the isolation measure is fully implemented (18).

Whilst many European countries and a number of states in the US are considering imposing further lockdowns to effectively tackle the COVID-19 crisis, an underlying question as to global pandemic governance relates to the timing of adopting preventive and interventionist measures, i.e., when the national health authority should implement such radical P&C measures as city or even region-wise lockdown together with rigorous isolation & confinement policies. In this paper, we provide a retrospective estimate of the scale of the pandemic spread under different scenarios of variation in key influencing parameters with a hybrid model. We have developed a new hybrid model of infectious disease transmission based on a configured epidemiology SIR (Susceptible-Infected-Recovered^5^) model coupled with a logistic probability function to analyze the COVID-19 outbreak and estimate its transmission pattern. A probabilistic contamination network is embedded in the pandemic transmission model to capture the randomness feature of person-to-person spread of the novel virus. Then an improved BP-SIR (Back Propagation-SIR) model is used to quantify the population contact state with isolation measures under different continuous time series contact probability. Further, the modeling parameters are adjusted to verify the model performance in accordance to the data from the reports published by various national governments and international organizations, including Centers for Disease Control (CDC) and the public health authority.

To account for the uncertainty in relation to the timing of health policy intervention in terms of consequences of infectious contamination and mortality, we have simulated two contrasting scenario groups in this paper. The first business-as-usual (BAU) scenario represents a trajectory where the government decides to maintain their modestly aggressive containment policy which reflects the *status quo* of the epidemic development in these countries during the early stage of outbreak, whereas another stringent policy scenario describes a non-delay or immediate policy response by assuming proactive P&C measures adopted and implemented effectively from the very beginning of an epidemic outbreak. Furthermore, the consequences of postponing stringent P&C policies are simulated by varying the delay in activation from 0 to 4 weeks to assess the varied consequences in terms of damage. This allows us to illustrate the importance of intervening in a timely manner in accordance with an optimal timing strategy. A delay in deciding to take strong action at an early stage would result in postponed arrival of the inflection point which in turn would delay the eventual control of virus transmission and exacerbate global pandemic spread.

To illustrate the epidemic growth pattern, our modeling will primarily focus on local dynamics of each epidemic event in a specific place in contrast with the pandemic, which resulted from spreading of the disease worldwide. The utility of this modeling logic is two-fold. (1) The careful examination of a local epidemic in a country is a prerequisite of understanding the underlying mechanism of the formation of a COVID-like pandemic; and (2) It allows us to draw useful lessons from inter-country comparison in terms of appropriateness of epidemic control measures and timing of government intervention in the light of better coordination when facing a global public health crisis, which is a sine qua non-condition of effective governance of a highly contagious epidemic which is further complexified by international movement and the context of globalized and interconnected economy. This study selected two subgroups as the modeling base, i.e., developed countries (US, Italy, Germany and France) and developing countries (India, Brazil and China), given the fact that all of them were heavily struck by the coronavirus, whereas markedly different outcomes were produced over the course of pandemic development.

Our modeling results indicate that early-stage preventive measures are the most effective way to contain the pandemic spread. In addition, appropriate state interventions in macro-management of human and socio-economic resources, i.e., enforcing social distancing and quarantines, and setting up special hospital facilities, are all essential to constrain the global transmission of the virulent infection. With the rapid spread of the novel coronavirus worldwide, this pandemic is no longer a single country's affair, but is on the way to develop into a global security concern requiring cooperation and control of all countries (19). To do so, internationally coordinated actions are required through sharing good practices.

## Methods

A number of prior studies have addressed this issue around prevention effectiveness by using different modeling paradigms (20, 21), many of them employed conventional bio-mathematical modeling strategies that describe the dynamics of the spread of infectious diseases such as the Susceptible-Exposed-Infected-Removed (SEIR) model (22) or stochastic transmission model (23). However, few of them have integrated the probabilistic approach into SEIR modeling consideration. Our modeling strategy is concisely described as follows.

First, the BP network is used to train the model which is a regression problem, where we use China's case as the baseline model. The model inputs are the active infected cases and death rate in China from February 15, 2020 to March 15, 2020, here, we define: total affected cases = active affected cases + removed cases, removed cases = death + recovered. We select the fitted objective as the infection rate from the susceptible state (*S*) to the infected state (*I*). The transition state from the infected to the removed is defined as a constant variable since isolation is the main concern which has little influence on the death and recovery of patients. The estimate of the infected cases is obtained with the combination of the SIR model and the calculation of the infection rate from each iteration, then the loss function is calculated as the mean squared error (MSE) between the estimate and actual infected cases. The mathematical expression of the infection rate is expressed by a logistic function:

(1) $$\begin{array}{c} RateSI=\frac{ce^{-\alpha (t+bias)}}{{\left(1+e^{-\alpha (t+bias)}\right)}^{2}} \end{array}$$

where *α* is the rate of change of *RateSI*; *bias* is used to select the starting point of the function variation. According to the real data, set bias = −10, and *c* is the initial value of *RateSI*.

**Figure 2:**
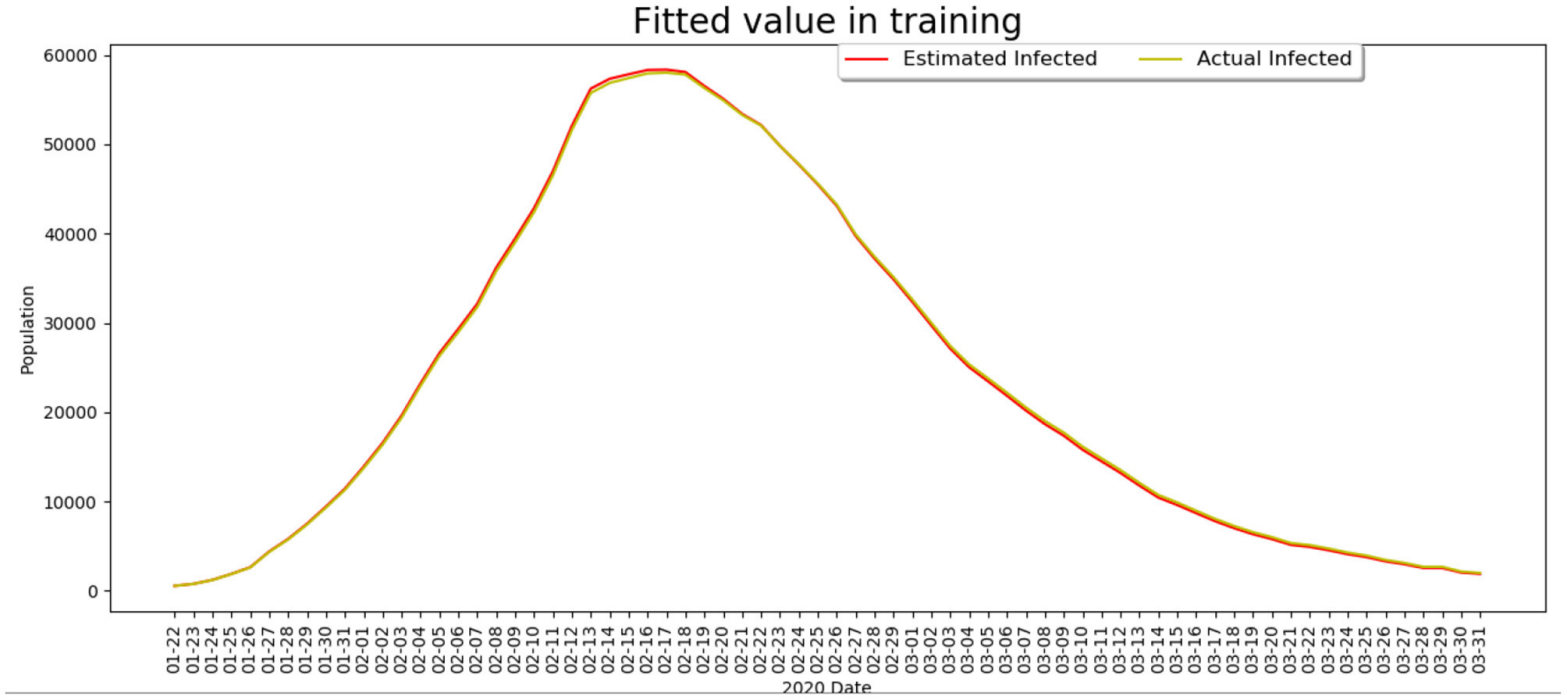
The comparison of the infected cases between the model estimate and the real data.

To make the model realistic and interpretable, we first calibrate the data of China to fit the model such that key parameters in Equation (1) can be derived. We use the real data from February 15, 2020 to March 15, 2020 in China published by WHO to train the BP-SIR model, and the fitted model estimate is compared with the actual data, shown in Figure 2. It can be seen that the developed model can well-predict the virus transmission situation.

Two prevention control strategies are used as follows:

1. Business as usual or delayed P&C policy, adopted by the government currently (issued on March 10, 2020), which is embodied in the *α*, *bias*, and *c* parameter settings for modeling prediction.
2. Stringent policy case, where countries began to take strict quarantine measures similar to Hubei Province, China from March 16, 2020. In the model, the values of α and *bias* are set based on the situation of Wuhan from January 23 to February 28, and the forecast days are dependent on the days of delay in the “delay” experiment (i.e., the sum of days to predict and the delay days is equal to the time span of the whole propagation process), while the other parameters are kept unchanged (strict quarantine).

**Figure 3:**
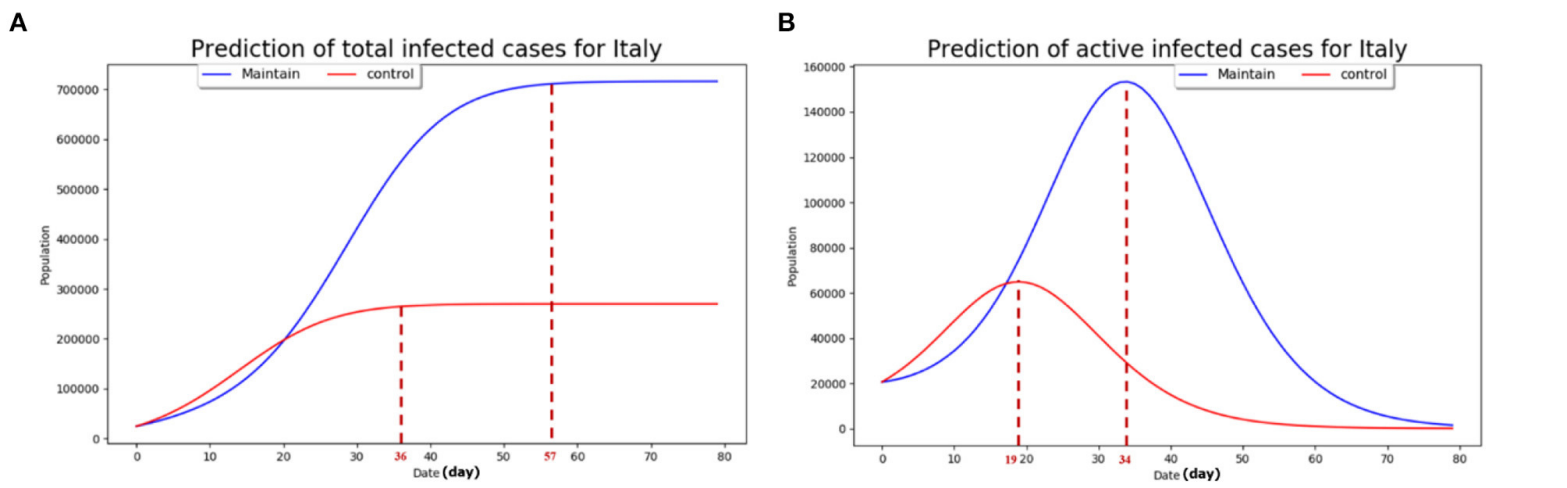
The predictions based on the developed model. **(A)** The estimate of the total infected cases with the two prevention control strategies; **(B)** the estimate of the current infected cases with the two prevention control strategies. *The maintain* scenario refers to the case where the public authority's mobilization of social resources to tackle the epidemic spread is relatively slow and inefficient with disobedience of the general public. The *control* scenario refers to the case of strict prevention and control measures adopted by the government at the initial phase of the COVID-19 outbreak with good obedience by the general public with regards to mandatory regulation such as lockdown.

It can be seen from Figure 3A that the total affected cases would be stable after 57 days (day 0 in Figure 3 refers to March 15, 2020), while the epidemic situation can be stabilized after 36 days with strict quarantine measure being taken similarly to Hubei province in China. Thus the number of the total infected cases with BAU isolation measures is about three times of that with strict measures while achieving stable status.

From Figure 3B, it can be seen that the active infected cases will reach peak at the 34^th^ day (roughly 65,000) and the 19^th^ day (roughly 155,000), the so called “inflection point,” with current isolation measure adoption and strict quarantine measure adoption similar to Wuhan district, respectively. The peak value of the active affected cases in the BAU epidemic control strategy is about 2.5 times higher than that under the strict prevention control strategy. We used the real data from March 15, 2020 to March 18, 2020 to test the developed model and the estimate results with active infected cases and total infected cases. Based on this baseline model, we present in the next section the projected infection cases in four major countries which experienced sharp increases since the widespread outbreak outside China in early March, namely the US, Italy, Germany, and France.

## Results and Discussion

**Figure 4:**
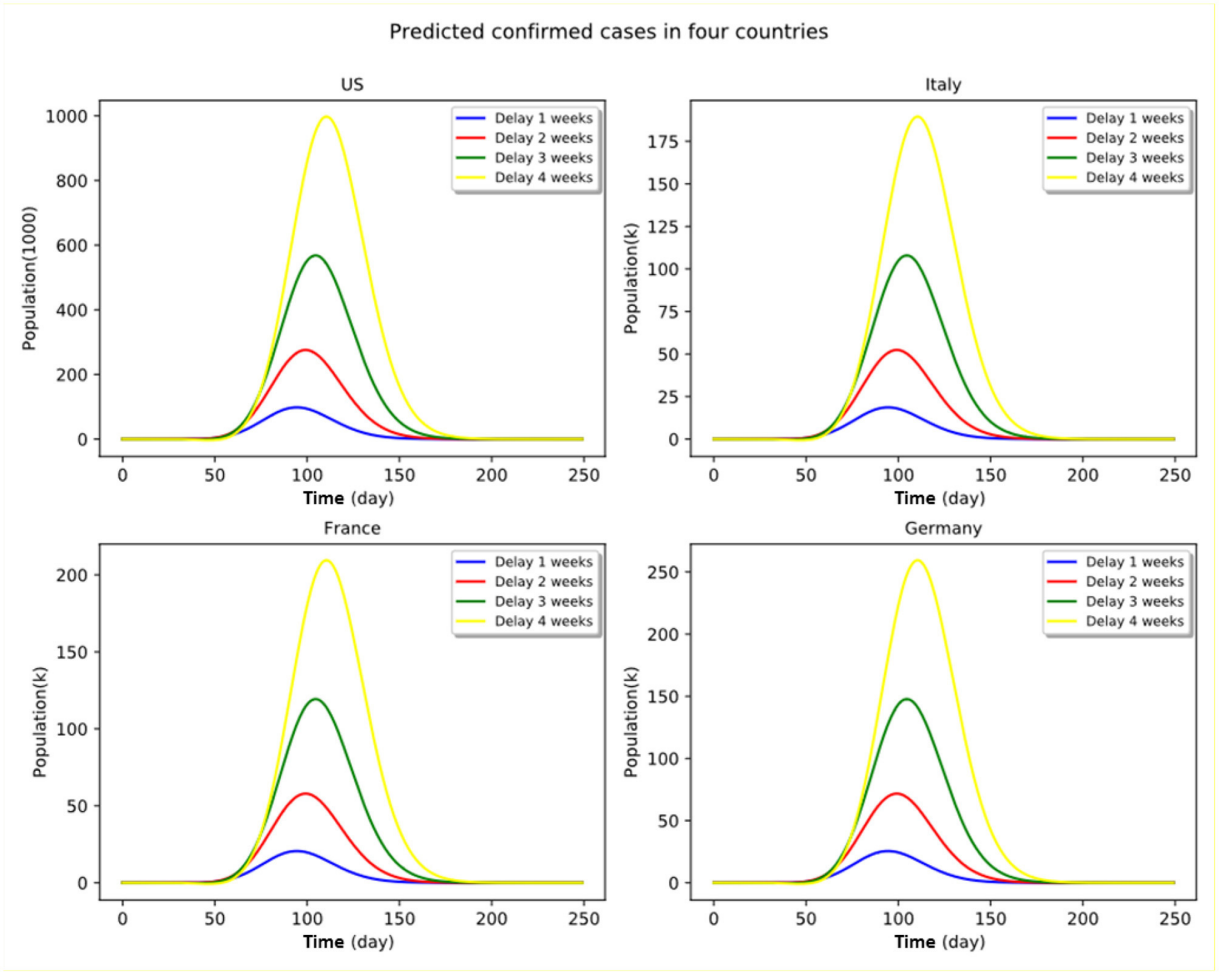
Proactive vs. delayed implementation of P&C measures under stringent policy. Numbers shown in the panel graphs are relative to the no-delay cases.

The modeling results of the infection cases and deaths caused by COVID-19 in the four countries under four counterfactual “delay” scenarios with the assumption that the number of the infected at the early stage of the epidemic is relatively small, whether in a country with a large population or with a small population, are presented in Figure 4. We assumed in the model that for each country, a 2-week observation time, starting from the initial outbreak in the country, is allowed for the government to make decisions about when to take such strong P&C actions as travel restrictions and large-scale lockdown, any further postponed actions in making such decisions will be considered as a delayed intervention in controlling the spread of the pandemic, spanning from 1 to 4 weeks.

It can be clearly seen that the timing *per se* is a critical factor in shaping the infection and death curves across all countries affected by the coronavirus outbreak, regardless of whether the government has decided to take strong (e.g., city lockdown and public space shutdown in Northern Italy) or moderate actions (e.g., herd immunity strategy initially adopted by the UK government) at the initial stage of the outbreak.

In other words, if a government envisages taking strong policies to tackle the COVID-19 epidemic, the decision of whether it chooses to implement the policy immediately or to delay in activating stringent P&C measures such as city lockdown and mandatory self-quarantine, may lead to stark differences in epidemic spread in terms of number of infections and deaths. Delaying the epidemic control policy may result in irreversibly large impacts on the infected countries as a result of the overwhelming peak of the number of infections and saturated medical resources. For instance, in the case of a strong policy scenario in France, doubling the delay period (from 2 to 4 weeks) of implementing the stringent quarantine measures would increase the peak number of active infection cases by a factor of more than four.

Under the current circumstances of rapid transmission of the coronavirus disease, all the countries in the world are obliged to take strong actions to minimize the irreversible damage and to avoid a global heath calamity. As such, taking a strong policy as early as possible is an optimal strategy as any delays may result in irreversible damages. The epidemic distribution curves may be effectively flattened by timely intervention of public authority coupled with stringent P&C measures. The peak infections would increase exponentially if the immediate subsequent weeks following the outbreak were forgone without an appropriate pandemic control policy. Stringent P&C measures may allow all countries to significantly reduce both infected cases and number of deaths caused by COVID-19 across all four countries. However, in the case of significant delay, the stringent policy measures are less likely to flatten the infection cases' distribution curves.

Our hybrid modeling results clearly indicate the paramount importance of taking timely preventive measures in public health systems when dealing with such a contagious pandemic as COVID-19 given its high person-to-person transmission risk, as disastrous consequences would be generated if the optimal window of intervention were forfeited at the early stage of outbreak, in particular in densely populated areas (i.e., the intra-network transmission probability may be increased exponentially) with scarce medical resources. It is of paramount importance to cut off virus contamination channels through social networks and interpersonal spread at the beginning of epidemic outbreak.

In particular, timing is more important than the degree of policy stringency *per se* in accelerating the arrival of peak of infection (or the so-called inflection point). In other words, the positive effects of stringent P&C measures would be canceled out in the case of significantly delayed action, whereas a moderately delayed control policy may still be able to contribute to containing the degree of epidemic spread although its effectiveness may be significantly compromised compared to a scenario of early intervention coupled with stringent P&C measures. A procrastinatory activation strategy adopted by the government and health authority to deal with the uncertainly of COVID-19 pandemic development at the early stage of outbreak turned out to be a worse strategy from the optimal control perspective, as significant irreversible damage would be produced in this case.

More importantly, early actions can significantly help flatten the shape of distribution of contamination. This will allow society to reduce transmissibility and severity which are the two most critical factors that determine the effect of an epidemic (24). The more a country delays implementing proactive P&C measures, the heavier the irreversible damage is in terms of infection and mortality (25).

**Figure 5:**
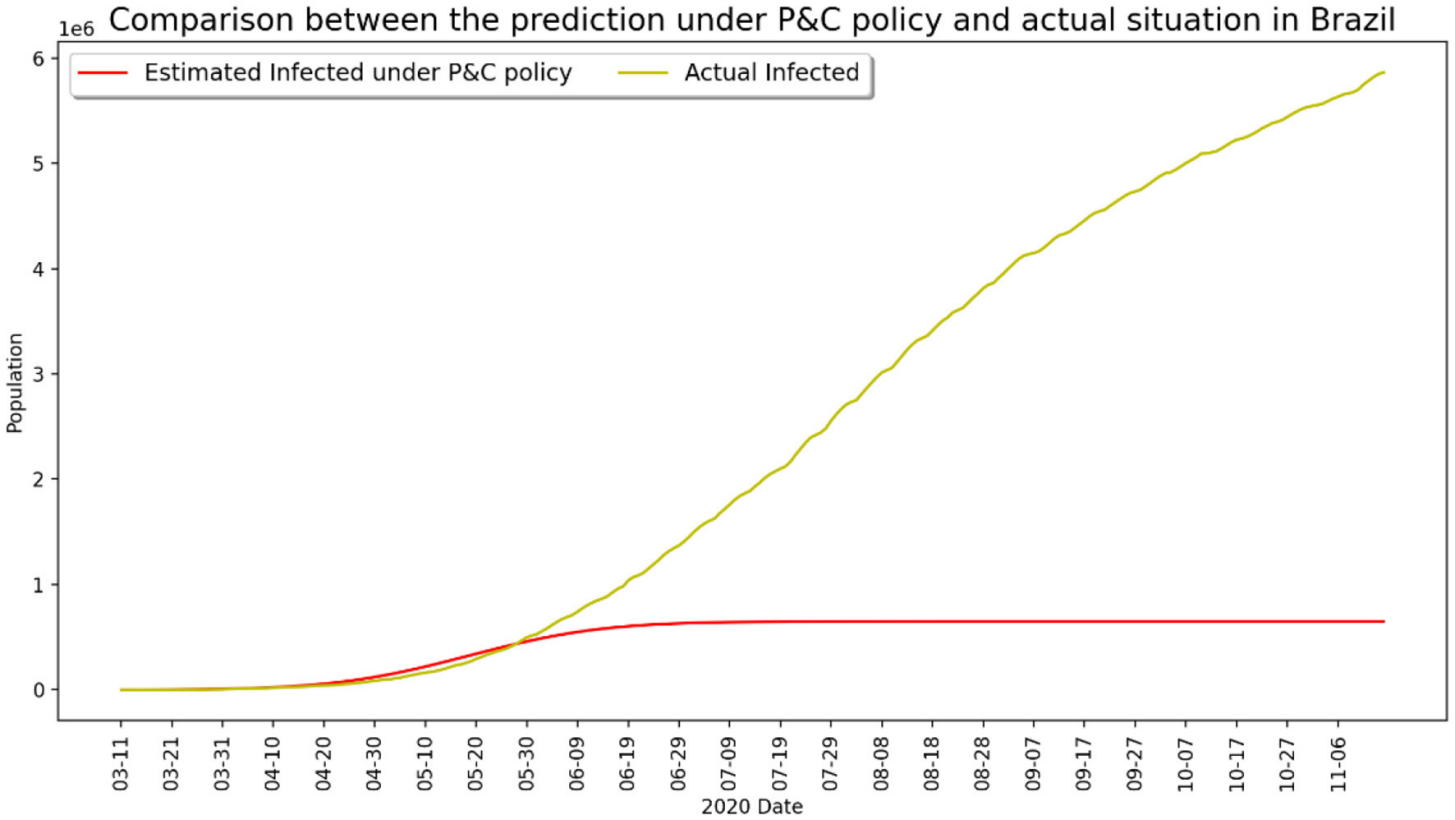
Infected number under strict P&C and actual situation in Brazil.

**Figure 6:**
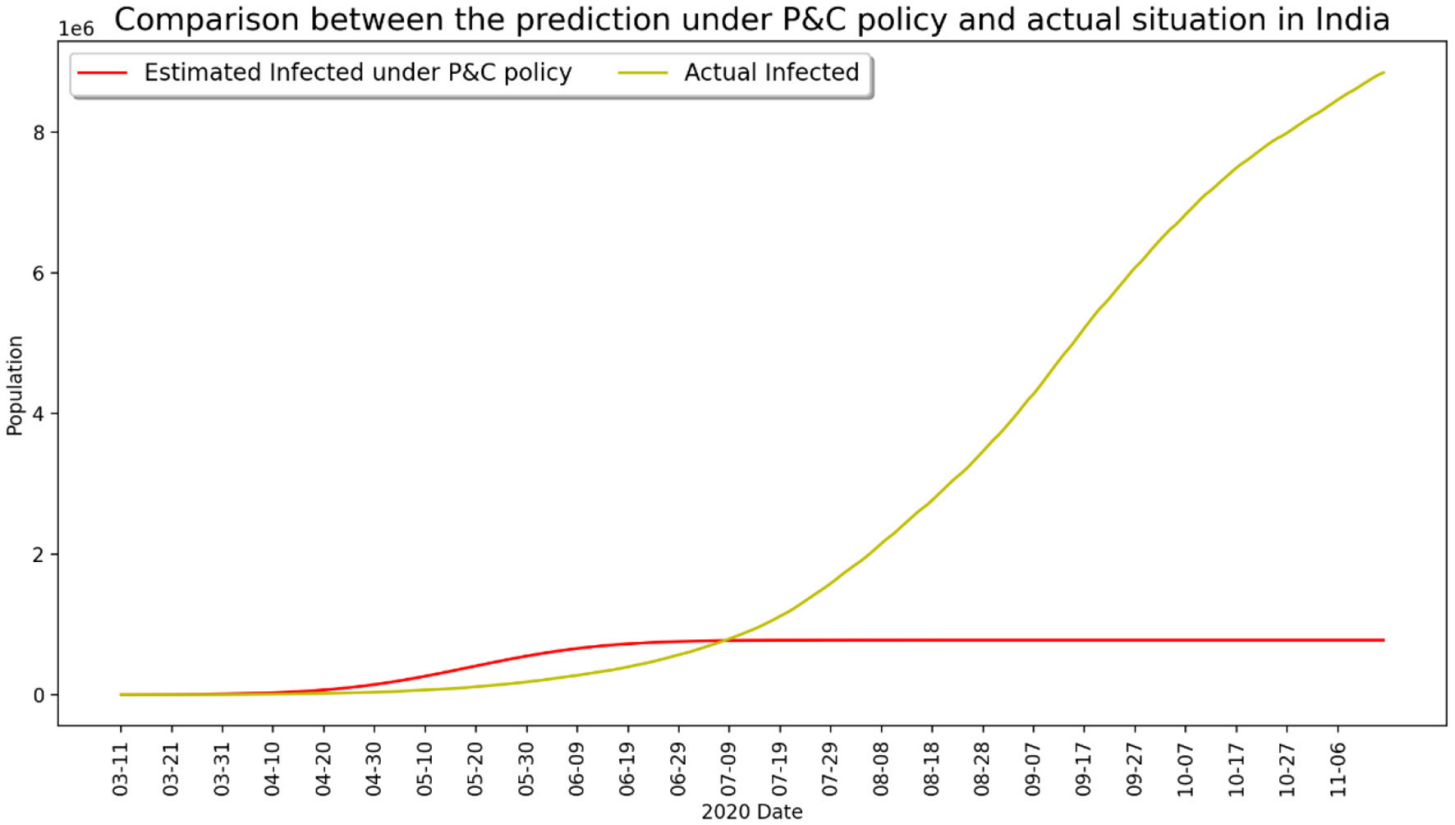
Infected number under strict P&C and actual situation in India.

Another painful lesson from the coronavirus pandemic is that several large-population nations have been seriously impacted due to weak state intervention and loose control of interpersonal contact during the early stage of epidemic outbreak, and the situation worsened after the global pandemic as a result of saturation of medical resources and unchecked disobedience of lockdown measures. Typical examples are Brazil and India, both experienced a large number of deaths over the course of pandemic development even after vaccination became widely available in early 2021. Figures 5, 6 present two contrasting scenarios in both Brazil and India, where it can be clearly seen that millions of lives would have been saved if the government had taken strict control measures and if the public had abided by the lockdown guidelines earlier. The recent disastrous situation in India further evidenced this observation.

## Policy Implications

From a retrospective viewpoint, the modeling findings of this research may offer some important implications for global public health governance, in particular for the cooperation and coordination of epidemic containment measures in the most vulnerable countries with weak medical responses to COVID-19-like global pandemic spread. The would-be outbreak in African and middle-eastern countries may draw some useful lessons from the major affected countries and regions, in particular the US and European experiences (26) in terms of optimal timing, i.e., activating emergency response mechanisms and adopting mandatory quarantine measures with close monitoring of the epidemic developments within and out of boundaries at the early stage of the outbreak. Any delay in implementing stringent P&C measures would produce irreversible disastrous consequences, posing severe threats to national and global health security and public welfare.

The main purpose of preventive intervention is to eliminate or minimize the source of infections, to cut off the route of transmission, and to protect the susceptible population, i.e., reducing public gatherings and close interpersonal contact, good hygiene practice such as wearing masks outdoors and washing hands frequently, canceling entertainment, social and religious gathering activities, limiting traffic and mobility, strengthening quarantine at transportation hubs such airports, railways, and bus stations, temporarily closing schools and public places, and carrying out thorough disinfection when necessary (27). In addition, to increase the efficiency of isolation of the infected population, early detection, diagnosis, and treatment are highly recommended, including routine temperature measurement of the vulnerable population, screening and monitoring of fever patients, and centralized isolation of the suspected cases and confirmed patients. In the event of a high risk of contamination, more draconian quarantine measures such as home confinement of entire infected areas with tight mobility restriction should be implemented (such as the case of lockdown in Wuhan and Northern Italy) (28).

Italy, as the second most seriously affected country (death toll in Italy is ranked the first in the current world situation) by the pandemic outside of China at the early stage of the coronavirus outbreak, has taken strict prevention measures similar to the Chinese pattern, i.e., closing down seriously infected towns and regions in order to take control of interpersonal spread. However, the current situation is far from being controlled effectively due to non-strict implementation of confinement and lack of stringent measures, such as home isolation and total suspension of urban mobility. Since the confinement started a week ago, it is reported that people are still moving around within the isolation zones, and cost-efficient self-protection measures such as face mask wearing have not been generalized to minimize the risk of person-to-person transmissions through respiratory airways. In addition, the situation was further complicated by the overload on local health systems due to a sharp increase in infected people in a short period of time and local medical capacity soon becoming saturated. The national intervention efficacy may not sustain without timely external support from third parties such as the European Union. The comprehensive mobilization and support pattern during the Wuhan Crisis at the beginning of February in China may provide a useful blueprint of efficient intervention for both the European Union and Italian decision-makers.

**Figure 7:**
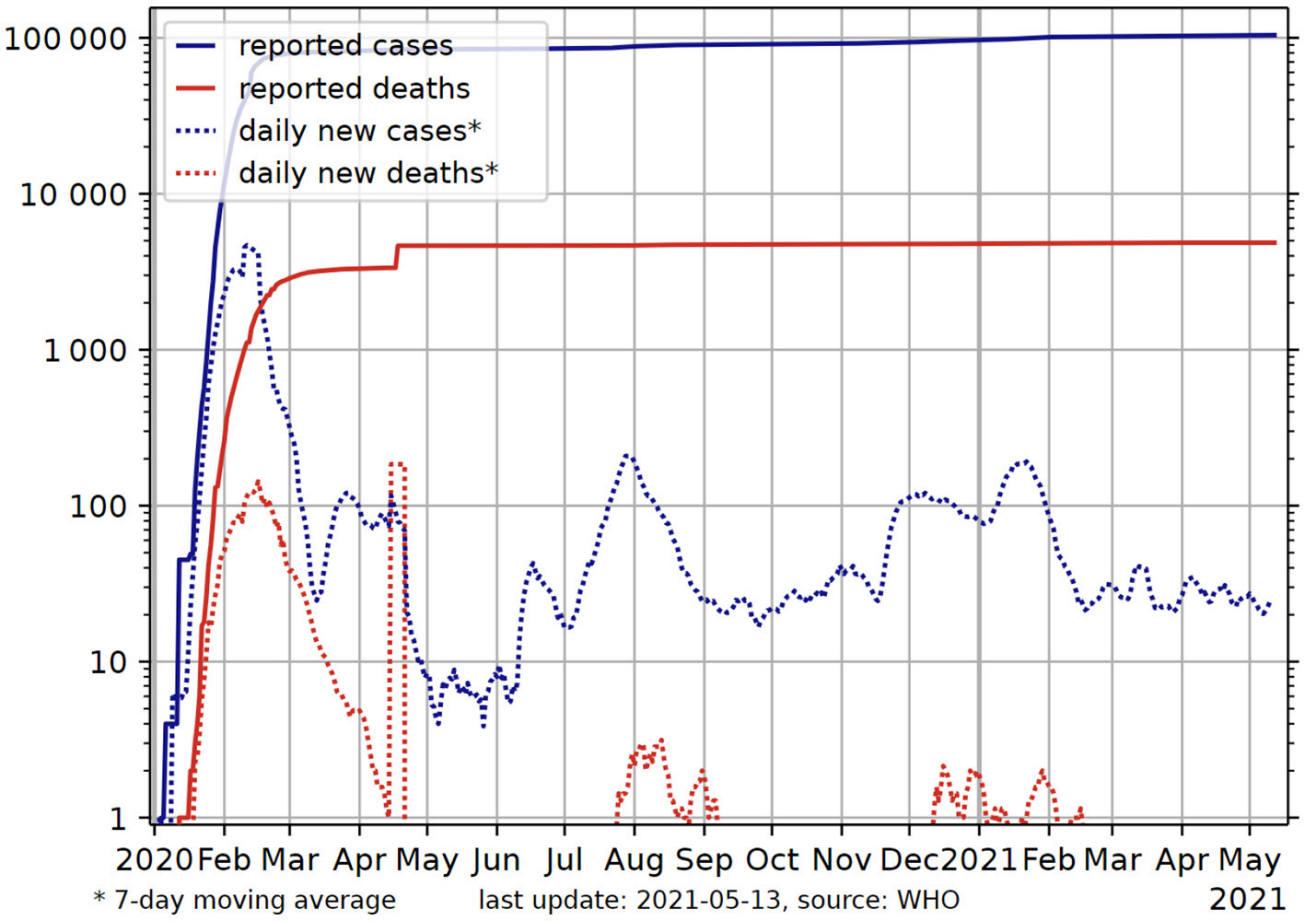
Total confirmed cases and deaths in China from early 2020 to date. Source: World Health Organization (29).

In addition, the effective containment of COVID development relies heavily on whether the people in a concerned country respect the guidelines and recommendations provided by the public health authority and scientists, e.g., wearing mask, social distancing, avoiding public gatherings, and respecting curfew and lockdown instructions. Such ignorance and disobedience behavior should be seriously avoided, otherwise the situation may become critical and the infected number could increase out of control in a very short period of time. The wild spread of the disease in the US and Brazil after the pandemic and the recent human tragedy produced in India demonstrate the importance of regulating individual conduct, and that the social costs of tolerating anarchical individual freedom can be astronomical. In comparison, the rigorous compliance of the public with the government P&C guidelines has allowed China to successfully cope with the epidemic since the outbreak. The growth of total confirmed cases and reported deaths in China was brought under control since the Wuhan lockdown was lifted in April 2020 and remains relatively stable over the last 12 months, as shown in Figure 7.

However, it is worth mentioning that adapted economic policy measures have to be put in place to accompany the stringent P&C implementation to facilitate people's obedience without losing their jobs or sacrificing family income, and specific financial resources or funds must be mobilized to provide a minimal surviving condition for those who struggle in isolation. Strict measures such as lockdown may face legitimate challenge if people's basic daily needs in impacted areas cannot be duly addressed. The lack of financial and social support was the primary reason why countries like Mexico, Brazil, India, and most of Africa have not succeeded in any effective lockdown policies so far. The government could choose to move directly into what should be done to assure an early global lockdown, and the coordinative and obedient action could save both lives and money^6^. In this regard, the multi-level coordination and cooperation between both state and sub-state actors is of crucial significance.

Research efforts from both public health institutions and research organization across the world need to be effectively coordinated in order to provide timely advice to WHO and national governments, while the latter must be clear-eyed and sober about the underlying pattern of pandemic spread dynamics and possible evolution, and calibrate our policy and strategy accordingly. In this regard, effective isolation and quarantine measures can help minimize the uncontrolled spread of virus in the early stage of outbreak. For instance, in Singapore, anyone who is subject to a mandatory stay-at-home notice (usually a 14-day period of home isolation) may face severe judicial punishment if she/he violates the notice's requirement. Likewise, the similar experience in Wuhan, the epicenter of COVID-19, shows that the city lockdown policy brought significant benefits in terms of reducing new confirmed cases and mortality only after strict traffic control and compulsory isolation P&C measures came into effect 2 weeks following the lockdown notice on January 23.

In order to optimize the efficacy of pandemic prevention and control measures, implementation guidelines need to be formulated for effective infectious disease control, while coordinating personnel from health care, public security, transportation, and social service departments of the local community. An integrated resources management and allocation system needs to be established to allow local medical and social workers to jointly complete the work of screening and controlling the sources of infection. The success of joint implementation of the pandemic control guidelines depends upon the response of emergency planning such as cutting off the route of transmission and protecting the susceptible population with isolation and treatment of infectious diseases (30, 31).

## Data Availability Statement

Publicly available datasets were analyzed in this study. This data can be found here: who.int.

## Author Contributions

JL and YZ conceived and designed the study. JL and LY set up the experiments. LY and YZ collected the data and ran the model. JL, YZ, and JZ analyzed the data. JL, YZ, LY, and ZC interpreted the results and wrote the manuscript. All authors contributed to the article and approved the submitted version.

## Conflict of Interest

The authors declare that the research was conducted in the absence of any commercial or financial relationships that could be construed as a potential conflict of interest.

## Publisher's Note

All claims expressed in this article are solely those of the authors and do not necessarily represent those of their affiliated organizations, or those of the publisher, the editors and the reviewers. Any product that may be evaluated in this article, or claim that may be made by its manufacturer, is not guaranteed or endorsed by the publisher.

## Footnotes

^1^Taking global financial market responses as an illustrative example, the NSE's DW index lost more than 2,000 points twice in a week as coronavirus fears accelerated (CNBC, 12-03-2020). Oil prices collapsed by more than half and fell to their lowest level in 17 years (Financial Times, 18-03-2012).

^2^ https://fightcovid19.hku.hk/hku-documented-the-worlds-first-case-of-covid-19-reinfection/

^3^ https://www.nytimes.com/2020/03/13/opinion/china-response-china.html?0p19G=3248

^4^Majority of the new infections in China are imported cases now.

^5^In the classic epidemiology modeling literature, this is also commonly referred to as “Removed.”

^6^We thank an anonymous referee for pointing out this important issue.

## National Policies in Response to the COVID-19 Outbreak

**WHO COVID-19**https://www.who.int/emergencies/diseases/novel-coronavirus-2019.

**France**:

Ministère des Solidarités et de la Santé (solidarites-sante.gouv.fr);

Agence francaise de press https://www.afp.com/fr/.

Reuters https://www.reuters.com/article/us-health-coronavirus-france/france-extends-lockdown-to-april-15-as-coronavirus-wave-swamps-paris-idUSKBN21E1AT.

**UK**:

UK government https://www.gov.uk/.

Department of Health and Social Care

https://www.gov.uk/government/organisations/department-of-health-and-social-care.

**USA**:

The White House www.whitehouse.gov.

Centers for Disease Control and Prevention (CDC) www.cdc.gov.

CNN: https://edition.cnn.com/.

**Italy**:

Ministero della Salute (www.salute.gov.it);

http://www.protezionecivile.gov.it/attivita-rischi/rischio-sanitario/emergenze/coronavirus.

**Spain**:

Ministerio de Sanidad, Consumo y Bienestar Social

www.mscbs.gob.es

https://www.rtve.es/noticias/coronavirus-covid-19/

**Germany**:

Bundesgesundheitsministerium

https://www.bundesgesundheitsministerium.de/.

**China**:

Central government of the People's Republic of China (www.gov.cn),

China CDC (www.chinacdc.cn);

China National Health Commission http://www.nhc.gov.cn/.

**Iran**:

The Ministry of Health and Medical Education (MOHME)

http://ird.behdasht.gov.ir/.
